# Docosahexaenoic Acid, a Key Compound for Enhancing Sensitization to Drug in Doxorubicin-Resistant MCF-7 Cell Line

**DOI:** 10.3390/nu15071658

**Published:** 2023-03-29

**Authors:** Sergio Crovella, Allal Ouhtit, Shaikh Mizanoor Rahman, Md Mizanur Rahman

**Affiliations:** 1 Biological Sciences Program, Department of Biological and Environmental Sciences, College of Arts and Sciences, Qatar University, Doha 2713, Qatar; 2 Obesity and Cancer Biology Lab, Natural & Medical Sciences Research Center, University of Nizwa, Nizwa 616, Oman

**Keywords:** breast cancer, chemoresistance, natural bioactive compound, docosahexaenoic acid, chemosensitization, drug accumulation, apoptosis

## Abstract

Drug resistance is a well-known and significant obstacle in the battle against cancer, rendering chemotherapy treatments often ineffective. To improve the effectiveness of chemotherapy, researchers are exploring the use of natural molecules that can enhance its ability to kill cancer cells and limit their spread. Docosahexaenoic acid (DHA), a lipid found in marine fish, has been shown to enhance the cytotoxicity of various anti-cancer drugs in vitro and in vivo. While the combined use of chemotherapeutic drugs with DHA demonstrated promising preliminary results in clinical trials, there is still a significant amount of information to be discovered regarding the precise mechanism of action of DHA. As the biological pathways involved in the chemosensitization of already chemoresistant MCF-7 cells are still not entirely unraveled, in this study, we aimed to investigate whether DHA co-treatment could enhance the ability of the chemotherapy drug doxorubicin to inhibit the growth and invasion of MCF-7 breast cancer cells (MCF-7/Dox) that had become resistant to the drug. Upon treating MCF-7/Dox cells with DHA or DHA–doxorubicin, it was observed that the DHA–doxorubicin combination effectively enhanced cancer cell death by impeding in vitro propagation and invasive ability. In addition, it led to an increase in doxorubicin accumulation and triggered apoptosis by arresting the cell cycle at the G2/M phase. Other observed effects included a decrease in the multi-drug resistance (MDR) carrier P-glycoprotein (P-gp) and TG2, a tumor survival factor. Augmented quantities of molecules promoting apoptosis such as Bak1 and caspase-3 and enhanced lipid peroxidation were also detected. Our findings in the cell model suggest that DHA can be further investigated as a natural compound to be used alongside doxorubicin in the treatment of breast cancer that is unresponsive to chemotherapy.

## 1. Introduction

Breast cancer (BC) is responsible for the highest mortality rate among women worldwide, making it the deadliest cancer [1,2]. The incidence of BC in women is rapidly increasing, with 2.3 million new cases diagnosed each year, and over 680,000 deaths from BC recorded in 2020, according to the World Health Organization (WHO) [3]. Abnormalities and imbalances in cell proliferation and apoptosis signaling pathways have been identified as fundamental factors in BC progression [2,4,5].

One of the significant biomarkers for the onset of BC is considered to be the estrogen receptor beta (ER). Over 80% of BCs are known to be “ER-positive,” indicating that the growth of BC cells is dependent on estrogen. Additionally, approximately 65% of these BC cells are also dependent on progesterone, referred to as “PR-positive” [6,7,8]. BC is a heterogeneous disease classified into four primary subtypes, including ER/PR+ and Her2− with Luminal A; ER/PR+ and Her2+ with Luminal B; and ER/PR−, Her2+ and ER/PR−, Her2− with triple-negative/basal-like tumors [6].

Several cell lines have been routinely used for in vitro experimental studies of BC, particularly MCF-7 and MDA-MB-231 cells. MCF-7 cells belong to the Luminal A subtype, exhibit slower growth, and respond to both endocrine therapies and systemic chemotherapy. In contrast, MDA-MB-231 BC cells are highly aggressive, metastatic, and poorly differentiated triple-negative BC (TNBC) cells that do not express ER, PR, or HER2 [9,10].

Numerous synthetic and natural compounds have been investigated for their anti-BC properties in various cancer cell lines [2,11,12,13,14,15,16,17,18,19,20,21,22]. However, drug resistance remains a significant hurdle to their efficacy, emphasizing the need for novel targeted therapies [2,23]. The overexpression of efflux transporters and transporter-mediated efflux has been linked to multi-drug resistance (MDR) [2,24,25,26,27]. These transporters pump drugs out of cells, causing a decrease in intracellular drug concentration [28]. Chemoresistance development leads to tumor progression and aggressive phenotypes [29], thereby increasing the mortality rate of cancer patients.

Currently, there are various interventions aimed at sensitizing and enhancing the response of drug-resistant tumors. Doxorubicin is currently the most potent chemotherapy medication used in BC therapy [30]. It works by inhibiting DNA topoisomerase II, activating reactive oxygen species [31,32], and inducing cell death [33,34,35]. However, Doxorubicin-treated cells often show resistance due to its inability to accumulate in the nucleus and its reduced ability to induce DNA damage and apoptosis [33,34,35].

Several compounds have been developed and assessed for their potential in reversing drug resistance in tumors. One promising group of compounds are the N-3 long-chain polyunsaturated fatty acids (LCPUFA), specifically docosahexaenoic acid (DHA) and eicosapentaenoic acid (EPA), which have been shown to possess anti-tumorigenic properties [36,37,38,39,40,41,42,43]. In particular, DHA has exhibited more promising cytotoxicity effects than EPA at the same concentration in MDA-MB-231 and MCF-7 breast cancer cells [37,39].

Combining DHA with chemotherapy drugs has also shown promising results in various breast cancer cell lines [39,44,45,46], as well as in mice [40,46] and one human metastatic breast cancer trial [47]. However, the human trial, which has been ongoing since 2009, did not produce useful results for further translation into clinical practice.

Enriching cell membranes with DHA has been found to promote changes in membrane properties and membrane-mediated signaling pathways that elicit anticancer responses [48,49]. The n-3 LCPUFA content of membrane phospholipids is a crucial component of the dynamic and asymmetric cellular membrane that can enhance the cells’ ability to respond to chemotherapy [50]. Incorporating EPA and DHA into lipid rafts increases the clustering of large raft domains, which can act as mobile docking platforms to improve cell signaling transduction through protein/lipid trafficking, leading to increased cancer cell death [37,39,42,49,51,52,53,54,55,56]. The incorporation of DHA also disrupts lipid raft signaling, resulting in changes in cell proliferation, apoptosis, and survival [37,52,55].

Given the previous studies demonstrating the potential anticancer effects of DHA by reversing MDR, our research aimed to investigate the ability of the fish-derived DHA compound to reverse drug resistance and induce apoptosis in a widely used drug-resistant breast cancer cell model, MCF-7/Dox. Through this analysis, we aimed to provide insight into the molecular mechanisms by which DHA triggers apoptosis.

## 2. Methods

### 2.1. Cell Culture

The MCF-7 cells (ATCC, VA) or doxorubicin resistant MCF-7 cells (MCF-7/Dox) (a generous gift from Dr. Toshio Yoneda) were cultured in Dulbecco’s modified Eagle’s medium (DMEM) (supplemented with 10% fetal calf serum (FCS) and 1% penicillin/streptomycin combination) at 37 °C in a humidified incubator containing 5% CO_2_. Each experiment was performed in triplicate.

### 2.2. Cell Proliferation Assay

The MCF-7/Dox (Doxorubicin resistant) cells were seeded in a 96-well plate at a density of 2 × 10^4^ cells/100 µL per well. The cells were allowed to attach overnight and then treated with different concentrations of DHA, doxorubicin, alone, or in combination in fresh media. After 48 h of incubation, the culture medium was removed, and the cells were washed twice with PBS. The adherent cells were then stained with crystal violet staining solution (Cell Viability Assay Kit (ab232855), abcam, Boston, MA, USA) for 20 min, followed by washing and air-drying. The solubilization solution was added, and the absorbance was measured at 570 nm using a microplate reader as per the manufacturer’s instructions.

### 2.3. Invasion Assay

Approximately 2 × 10^4^ MCF-7/Dox cells were suspended in 200 µL of serum-free DMEM and added to the upper chamber of a 24-well BioCoat Matrigel invasion chamber with an 8 µm pore size membrane, which was then placed in the corresponding assay plate from BD Biosciences (Bedford, MA, USA)). The lower chamber was filled with 700 µL of DMEM containing 10% fetal calf serum (FCS) and varying concentrations of DHA, doxorubicin, alone or in combination. Following a 48 h incubation period, cells remaining in the upper chamber were removed, and cells that had passed through the membrane pores and reached the lower side were fixed with 10% formalin and stained with 0.1% crystal violet blue solution. The stained cells were manually counted to determine the number of cells that had migrated through the pores, as previously described [22].

### 2.4. Doxorubicin Accumulation

To evaluate intracellular doxorubicin levels, MCF-7/Dox, and parental MCF-7 cells (1 × 10^5^) were cultured with various concentrations of DHA overnight. The cells were then exposed to 2 µM doxorubicin in complete PBS media supplemented with 0.5 mM MgCl2, 0.7 mM CaCl2, and 0.1% glucose, and incubated at 37 °C for 1 h. After washing with PBS, the cells were treated with lysis buffer containing 0.1% Triton X-100. Fluorescence was measured using a Perkin Elmer LS-50B fluorescence spectrometer with an excitation wavelength of 470 nm and an emission wavelength of 595 nm, as previously described [57].

### 2.5. Cell Cycle Analysis by Flow Cytometry

Cell cycle analysis was performed using flow cytometry. Briefly, 1 × 10^5^ cells were cultured in a 6-well plate overnight, and the media were removed after 24 h. The cells were then treated as described above with DOX or DHA, individually or in combination for 24 h. After trypsinization and centrifugation at 3000× *g* for 5 min, the pellet was dissolved in ice-cold ethanol and stored at −20 °C until use. Prior to cell cycle analysis, the cell pellet was washed trice with PBS and incubated at 37 °C in the presence of RNase A. The cells were then stained with 0.5 µg/mL of propidium iodide for 30 min prior to FACS analysis (BD Biosciences- Allschwil, Switzerland).

### 2.6. Annexin V Apoptosis Assay

An Annexin V-FITC staining kit (BD Bioscience, Franklin Lakes, NJ, USA) was used to analyze apoptotic cell death. MCF-7/Dox cells grown in 60 mm diameter dishes were treated with either DOX or DHA alone or in combination for 24 h. After trypsinization and centrifugation, the cell pellets were washed with PBS and suspended in 100 μL of binding buffer containing 5 μL of Annexin V-Fluorescein isothiocyanate (FITC) and 5 μL of propidium iodide (PI). The stained cells were then incubated for 15 min at room temperature and analyzed using a FACSCalibur flow cytometry system (BD Bioscience, Franklin Lakes, NJ, USA).

### 2.7. RNA Isolation and Quantitative Real-Time RT-PCR

MCF-7/Dox cells were treated with 2 uM of DOX or 50 uM of DHA, individually or in combination for 48 h. Total RNA was isolated using Trizol reagent (Invitrogen) as described by the manufacturer. After DNase treatment, the RNA was transformed into cDNA using SuperScript™ III First-Strand Synthesis SuperMix (Invitrogen, Waltham, MA, USA). Relative gene expression was determined using QuantStudio™ 12 K Flex System (Applied Biosystems, Carlsbad, CA, USA) and GoTaq qPCR Master Mix (Promega, Madison, WI, USA). The primers required for RT-PCR analysis were obtained from Integrated DNA Technologies (Coralville, CA, USA). β-Actin gene expression analysis was used as an endogenous control and normalization was performed using the 2^−ΔΔCt^ method to set the values of control as one. The relative gene expressions of P-gp, TG-2, and apoptotic markers, in particular, Bak1 and caspase 3 were investigated in this study.

### 2.8. Lipid Peroxidation Assay

The effect of DHA in the presence or absence of DOX was examined in this study using Lipid Peroxidation (MDA) Assay Kit (ab118970). MCF-7/Dox cells (2 × 10^4^ cells per well) were cultured in a black 96-well plate and incubated at 37 °C in a CO_2_ incubator. The next day, the cells were treated with various concentrations of DHA (25, 50, and 100 uM) in the presence or absence of 2 uM of DOX. The cells were then incubated at 37 °C for 24 h. After removing the media, the cells were washed with PBS, incubated in TBA solution at 95 °C for 60 min, cooled in an ice bath for 10 min, and then analyzed with a microplate reader (Ex/Em = 532/553 nm) as described in the manufacturer’s protocol.

### 2.9. Statistical Analysis

The mean ± SE was used to express the values obtained from statistical analysis. A value of *p* < 0.05 was considered statistically significant by one-way ANOVA. The comparison between the means of groups was carried out using Newman–Keuls and Dunnett’s multiple-comparison tests.

## 3. Results

Anti-inflammatory bioactive fatty acids are well-known for their anti-cancer effect. To find an effective adjuvant bioactive compound to treat doxorubicin-resistant MCF-7 cells, we first checked for the anti-cancer effect of several known bioactive fatty acids against MCF-7 parental cells. Interestingly, among all tested compounds, DHA showed the best anti-breast cancer proliferative effect (Supplemental Figure S1). Based on this preliminary screening, we chose DHA to combine with doxorubicin to treat MCF-7/Dox. Further, to determine if the anti-breast cancer effect of DHA is cancer-specific, we also performed cytotoxicity experiments against normal breast epithelial cells, MCF-10A. We found that up to 100 μM of DHA is non-toxic to normal cells (Supplemental Figure S2). Our finding is also supported by other investigators’ findings [17,58].

**Figure 1:**
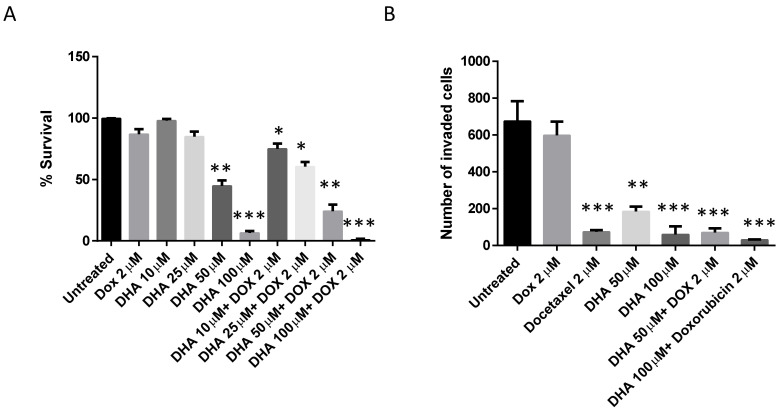
The impact of DHA on the proliferation, and invasion of doxorubicin-resistant MCF-7 breast cancer cells (MCF-7/Dox). (**A**) For the cell proliferation experiment, the MCF-7/Dox cells were treated with various concentrations of DHA, doxorubicin (2 µM) alone or in combination for 48 h, and stained using a crystal violet staining solution and measured the absorbance to determine the percent survival of cells. (**B**) For the invasion experiment, MCF-7/Dox cells in serum free medium were placed in the upper chamber of a 24-well BioCoat Matrigel invasion chamber and the lower chamber was filled with varying concentrations of DHA with or without doxorubicin (2 µM) in serum containing medium or with 2 µM of docetaxel alone as a positive control. After 48 h of incubation, the cells that traversed the membrane pores to the lower side were fixed with 10% formalin, stained with 0.1% crystal violet blue, and counted manually. The values shown represent the average ± standard error of the mean, obtained from three separate experiments. A *p*-value of <0.05 is considered significant by a Newman–Keuls one-way ANOVA with multiple comparisons test. * *p* < 0.05, ** *p* < 0.01, *** *p* < 0.001 compared to untreated control.

***Effect of DHA treatment on proliferation and invasion***: Cell viability assay was used to determine the effect of DHA co-treatment on the MCF-7/Dox breast cancer cell line. Our data demonstrated that DHA alone, dose-dependently, inhibited the MCF-7/Dox cell proliferation (Figure 1A). Doxorubicin treatment alone did not reasonably inhibit MCF-7/Dox. However, when doxorubicin was combined with DHA, these treatments significantly increased the inhibitory effect of Dox or DHA, using MCF-7/Dox cell proliferation of either treated culture (Figure 1A).

Metastasis is the ultimate fate of any advanced state cancer; once cancer is in its metastatic phase, it is manageable but not curable anymore. So, any treatment that can prevent metastasis would be an ideal candidate as a drug. Metastatic cancer cells require local intravasation, which involves passing through the extracellular matrix.

Moreover, to analyze the effect of doxorubicin and DHA alone or in combination, in the context of metastatic cell ability, we performed the BioCoat Matrigel invasion assay. Since the variant of MCF-7 cell is doxorubicin-resistant, we did not see any significant reduction in cancer cell invasion when treated with doxorubicin alone (Figure 1B). We treated the cells with another chemotherapeutic drug docetaxel as a positive control to see the comparative effect of DHA alone or in combination with doxorubicin. DHA in combination with doxorubicin dose dependently inhibited the MCF-7/Dox cell invasion as compared to either treatment alone (Figure 1B). These results indicate that a natural compound DHA could be a potential co-treatment strategy to minimize, in vitro, tumoral cell growth and migration of drug-resistant cancer cells. DHA co-treatment significantly reduced the invasion capacity of breast cancer tumor cells.

**Figure 2:**
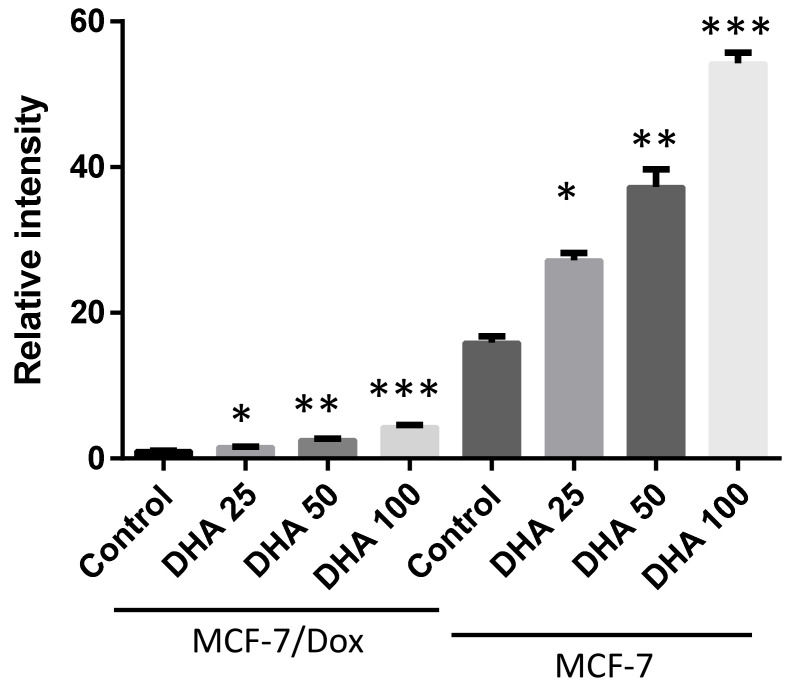
Effect of DHA on doxorubicin accumulation in doxorubicin-resistant and parental MCF-7 cells. Both Doxorubicin-resistant MCF-7/Dox cells and the parental Doxorubicin-sensitive MCF-7 cells were treated with various concentrations of DHA overnight, followed by treatment with 2 µM of doxorubicin for 1 h at 37 °C. After washing, cells were lysed using a lysis buffer, and fluorescence intensity was measured using a Perkin Elmer LS-50B spectrometer with an excitation wavelength of 470 nm and emission wavelength of 595 nm. The values presented are the mean ± standard error of the mean, based on three independent experiments. A *p*-Value of <0.05 is considered significant by a Newman–Keuls one-way ANOVA with multiple comparisons test. * *p* < 0.05, ** *p* < 0.01, *** *p* < 0.001 compared to respective untreated control.

***Effect of DHA co-treatment on doxorubicin accumulation in MCF-7/Dox cells:*** Drug sensitivity is known to increase with drug accumulation in tumor cells, while reduction in drug incorporation in cells is a key mechanism of drug resistance in tumors. When doxorubicin-resistant (MCF-7/Dox) and sensitive (MCF-7) cells were treated with doxorubicin alone, the drug accumulation in MCF-7/Dox was significantly lower than in MCF-7 cells (Figure 2). To investigate whether DHA co-treatment enhances cell killing by increasing doxorubicin in MCF-7/Dox cells, we analyzed the cellular doxorubicin content. Interestingly, DHA co-treatment significantly increased doxorubicin accumulation in MCF-7/Dox cells in a dose-dependent manner, possibly by altering the cell membrane composition (Figure 2). We also examined whether DHA co-treatment could enhance doxorubicin accumulation in parental MCF-7 cells. The doxorubicin accumulation was increased by 2-fold and 3-fold in the presence of 50 μM and 100 μM of DHA, respectively. Although DHA co-treatment significantly enhanced doxorubicin accumulation in both doxorubicin-resistant and sensitive MCF-7 cells, the doxorubicin accumulation was still lower in MCF-7/Dox cells than in parental MCF-7 cells (Figure 2).

**Figure 3:**
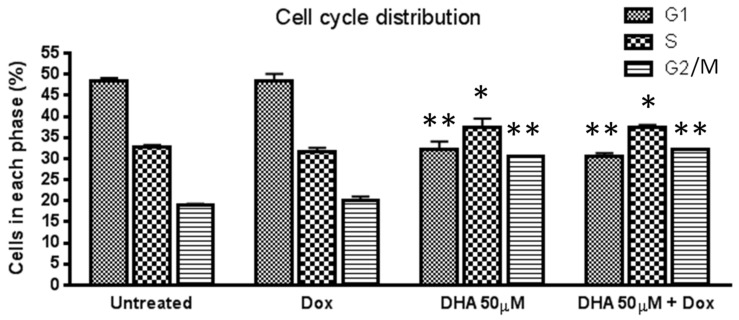
Effect of DHA co-treatment on cell cycle arrest in MCF-7/Dox cells. MCF-7/Dox cells were treated with either DHA, Dox, or a combination of both for 24 h, followed by isolation, treatment with ethanol, staining with propidium iodide, and FACS analysis to determine cell cycle distribution. The reported values represent the average ± standard error of the mean from three independent experiments. A *p*-value of <0.05 is considered significant by a Newman–Keuls one-way ANOVA with multiple comparisons test. * *p* < 0.05, ** *p* < 0.01 compared to respective untreated control. G1, Gap 1 phase for cell growth; S, synthesis phase for DNA synthesis; G2, Gap 2 phase for cell growth; M, mitosis phase for cell multiplication.

***Effect of DHA co-treatment on cell cycle arrest in MCF-7/Dox cells***: To investigate whether DHA co-treatment arrests MCF-7/Dox cells on a specific cell cycle phase, cell cycle analysis was performed using FACS. Treatment with doxorubicin alone did not significantly alter cell cycle distribution compared to the untreated control. However, treatment with DHA alone or in combination with doxorubicin resulted in a significant decrease in cells in the G1 phase and an increase in the G2/M phase compared to doxorubicin alone treated or untreated control. Additionally, DHA and DHA+Dox treatments increased the number of cells in S phase compared to doxorubicin alone treated or untreated control. These results suggest that DHA co-treatment increases the percentage of G2/M and S phase cells in MCF-7/Dox cells compared to untreated and doxorubicin alone treated cultures (Figure 3).

**Figure 4:**
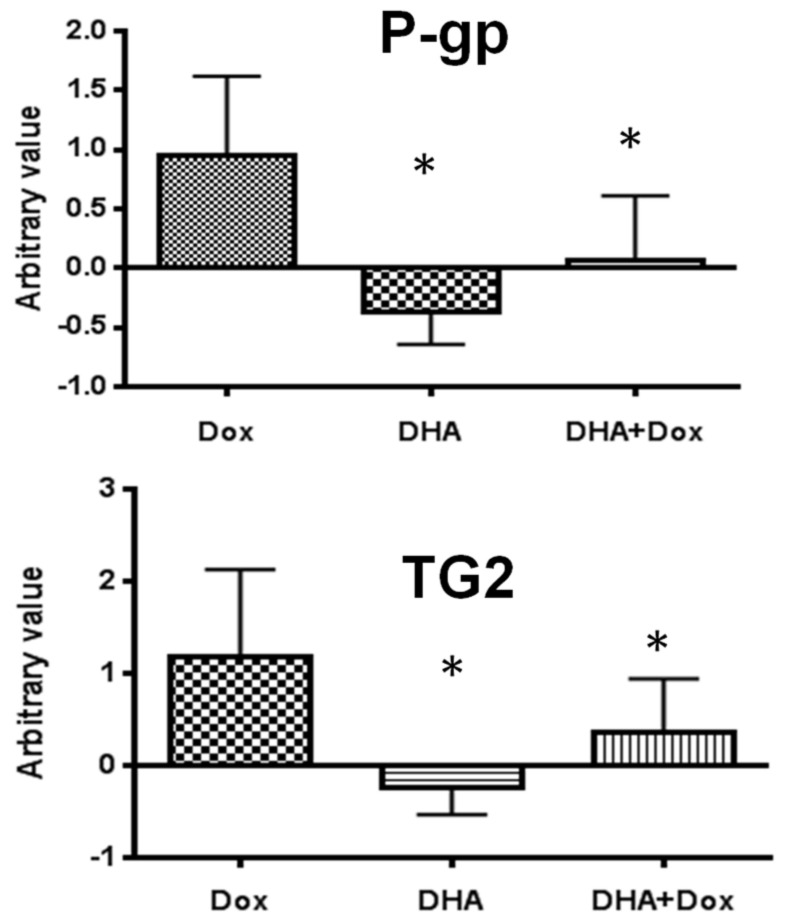
Effect of DHA on P-gp and TG−2 gene expression in MCF-7/Dox cells. MCF-7/Dox cells were treated with doxorubicin (Dox) (2 µM), DHA 50 µM, alone or in combination for 48 h. RNA was extracted and evaluated for gene expression through real-time RT-PCR. * A *p*-Value of less than 0.05 is considered significant when compared to the control group treated only with Dox, as determined by a one-way ANOVA.

***Effect of DHA co-treatment on the P-gp and TG−2 gene expression:*** P-glycoprotein (P-gp) is an ATP-binding cassette transporter that is strongly associated with multi-drug resistance in cancer cells. Transglutaminase 2 (TG−2), on the other hand, plays a critical role in shifting glucose metabolism, enabling cancer cells to survive in stressful conditions and enhancing their metastatic potential. To investigate whether DHA co-treatment can improve drug sensitivity by reducing the expression of these genes, we examined their mRNA levels using real-time RT-PCR. Interestingly, we found that DHA significantly down-regulated the expression of both P-gp and TG−2 genes (Figure 4).

**Figure 5:**
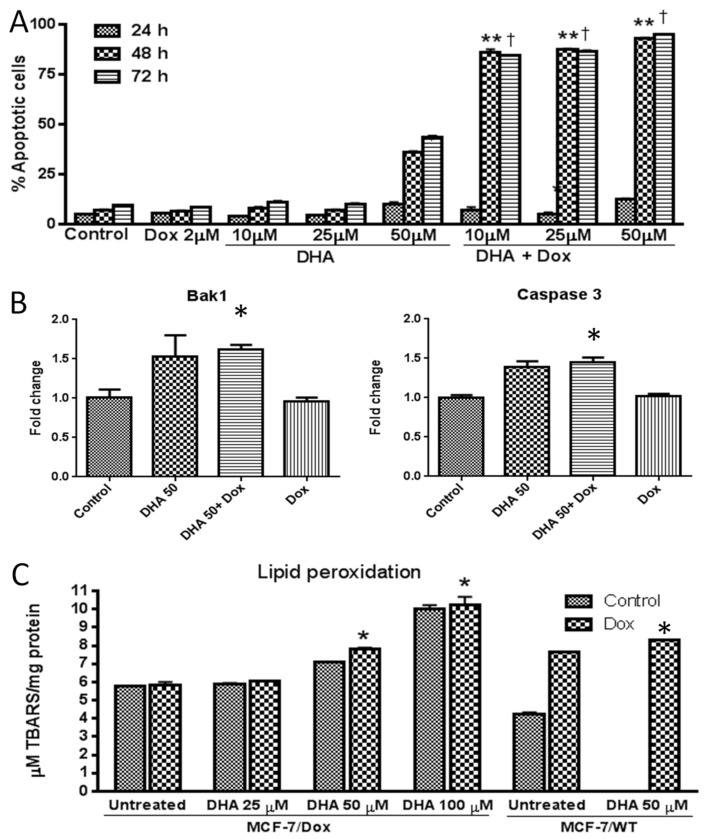
Effect of DHA on induction of apoptosis of MCF-7/Dox cells. (**A**) Percent apoptotic cell analysis by Annexin V staining and FACS, (**B**) Pro-apoptotic genes Bak1 and Caspase 3 measurement by real-time RT-PCR and (**C**) Assessment of lipid peroxidation using lipid peroxidation assay kit. The level of lipid peroxidation was studied in doxorubicin-resistant MCF-7 (MCF-7/Dox) as well as parental MCF-7 (MCF-7/WT) cells exposed to Dox only or a combination of Dox and DHA. Each value represents the mean ± SEM of independent triplicate cultures. A value of *p* < 0.05 is considered significant by a one-way ANOVA. ** *p* < 0.001, † *p* < 0.001, * *p* < 0.05 compared to respective Dox alone treated culture.

***Effect of DHA co-treatment in inducing apoptosis in MCF-7/Dox cells*:** Apoptosis induction is the main mechanism of action for most anti-cancer drugs. In this study, we aimed to investigate if DHA co-treatment can enhance apoptosis in doxorubicin-treated MCF-7/Dox cells, by Annexin V apoptosis assay using FACS analysis (Figure 5A). Our results showed that the combination of DHA and doxorubicin significantly increased apoptotic cell death, especially after 48 and 72 h of treatment. Furthermore, we examined the expression levels of pro-apoptotic genes, Caspase 3 and Bak1, by real-time RT-PCR (Figure 5B). Our data indicated that both genes were significantly up-regulated in MCF-7/Dox cells treated with doxorubicin in the presence of DHA, compared to cells treated with doxorubicin alone.

In addition, we investigated the role of lipid peroxidation in regulating cell death and also whether DHA co-treatment can enhance lipid peroxidation in doxorubicin-treated cells. We tested the levels of lipid peroxidation in parental (MCF-7/WT) and doxorubicin-resistant MCF-7 (MCF-7/Dox) cells. Our data showed that doxorubicin treatment alone increased lipid peroxidation in MCF-7/WT but not in MCF-7/Dox cells (Figure 5C). However, when combined with DHA, lipid peroxidation levels were significantly enhanced both in parental and doxorubicin-resistant MCF-7 cells (Figure 5C). Therefore, the combination of doxorubicin and DHA effectively enhances lipid peroxidation, not only in parental MCF-7 cells but also in doxorubicin-resistant MCF-7 cells, in a dose-dependent manner.

## 4. Discussion

**Figure 6:**
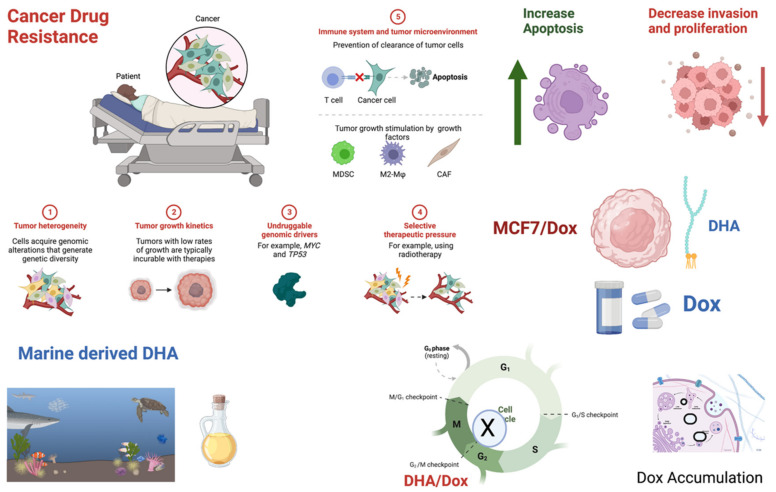
The effect of DHA administration on MCF-7/Dox cells.

Drug resistance is a significant problem in cancer treatment, which can develop through various mechanisms, including decreased drug uptake by cancer cells, increased drug efflux from cancer cells, and changes in drug target expression or function. DHA is a bioactive natural compound that has shown promising potential as a therapeutic agent for drug-resistant breast cancer. Several studies have demonstrated that DHA can sensitize breast cancer cells to chemotherapy drugs and overcome drug resistance [59,60,61,62]. However, the exact mechanisms by which DHA exerts its drug sensitization effects are still not entirely unraveled. In this study, we explored the possible mechanisms by which DHA exerts its drug sensitization potential. We found that co-treatment with DHA enhanced the drug sensitization of doxorubicin in already doxorubicin-resistant MCF-7 breast cancer cells by inhibiting cell proliferation and invasion, inducing G2/M phase cell cycle arrest, enhancing drug accumulation in cancer cells, inducing the expression of apoptotic genes, inducing lipid peroxidation, and down-regulating drug efflux-regulating genes such as P-gp and TG2. Figure 6 presents an overview of the potential mechanisms through which DHA may exert its anti-breast cancer effects.

N-3 long-chain polyunsaturated fatty acids (PUFAs), such as DHA, are recognized as potential agents to counteract invasive breast cancer and prevent cancer in animal models [50]. In rat models with a DHA-rich diet, DHA has been shown to act synergistically with doxorubicin and enhance its effectiveness against drug-resistant cancer cells. Several trials have investigated the effects of dietary supplementation with PUFAs in breast cancer patients (see https://clinicaltrials.gov/ct2/results?cond=breast+cancer&term=DHA&cntry=&state=&city=&dist= accessed on 26 December 2022), but few have reported positive outcomes. Therefore, additional preclinical studies are necessary to confirm the potential benefits of DHA and support its use in human trials.

Cell cycle progression is a tightly regulated process that is essential for the growth and division of cells. Recent studies have suggested that inducing G2/M phase cell cycle arrest can lead to the inhibition of cell proliferation and the induction of apoptosis [63,64]. In our study, we also found that DHA treatment, alone or in combination with doxorubicin, arrested the cell cycle of MCF-7/Dox cells at the G2/M phase. G2/M phase cell cycle arrest may be an effective strategy for sensitizing drug-resistant breast cancer cells to chemotherapy.

Cancer cells can become resistant to chemotherapy by various mechanisms including decreased drug uptake, increased drug efflux, altered drug targets, and increased DNA damage repair mechanisms. One of the key mechanisms of drug resistance is the reduction in drug accumulation within the cell [43]. Enhancing drug accumulation can overcome drug resistance and promote cancer cell killing. This can be achieved through various approaches such as co-treatment with agents that can inhibit drug efflux transporters, modification of the drug to improve its uptake, or alteration of the tumor microenvironment to increase drug penetration [65]. In our study, we found that doxorubicin accumulation was enhanced in both doxorubicin sensitive and resistant MCF-7 cells when treated with DHA. Downregulation of P-glycoprotein (P-gp) and Transglutaminase 2 (TG2) has been identified as a potential mechanism to sensitize drug-resistant breast cancer cells to chemotherapy [66]. These two proteins are known to be overexpressed in many drug-resistant cancer cells, leading to increased drug efflux and decreased drug accumulation, thereby reducing the effectiveness of chemotherapy [66,67,68,69,70]. One mechanism by which drug accumulation can be enhanced is by inhibiting drug efflux transporters, which are proteins that pump drugs out of cancer cells and decrease drug accumulation [70,71,72,73]. DHA was shown to increase the intracellular accumulation of drugs in cancer cells by altering the cell membrane composition [50] or inhibiting drug efflux transporters [22,74]. Our previous findings were supported by this study, which demonstrated that treatment with DHA resulted in down-regulation of P-gp protein expression in MCF-7/Dox cells [22]. However, the downregulation of these proteins may also have potential side effects and affect normal cell functions; thus, further studies are needed to fully understand the mechanisms and determine the optimal strategies for downregulating P-gp and TG2 in drug-resistant breast cancer cells.

Apoptosis and lipid peroxidation have been identified as potential mechanisms to sensitize drug-resistant breast cancer cells to chemotherapy. Apoptosis is a programmed cell death process that can be activated by chemotherapy, leading to the elimination of cancer cells. However, drug-resistant cancer cells often have defects in the apoptotic pathway, which allows them to survive despite exposure to chemotherapy. Our study demonstrated that treatment with a combination of DHA and doxorubicin resulted in increased expression of pro-apoptotic genes, leading to greater apoptotic cell death in MCF-7/Dox cells. Similar findings have been reported by others, who have shown that DHA can enhance the cytotoxicity of drugs against cancer cells by inducing the expression of apoptotic genes and activating apoptotic pathways [41]. Lipid peroxidation is a process where free radicals attack the polyunsaturated fatty acids (PUFAs) in cell membranes, resulting in the formation of lipid peroxides and other reactive oxygen species (ROS). This process has been linked to the development and progression of breast cancer and other cancer types. Studies have suggested that DHA may have an anti-breast cancer effect by inducing lipid peroxidation [75]. Recent research has indicated that ferroptosis may be a potential mechanism for DHA’s anti-cancer effects, particularly in breast cancer [76,77]. Ferroptosis is a regulated form of cell death characterized by the accumulation of iron-dependent lipid peroxides in the cell membrane, leading to oxidative damage and cell death. Studies have demonstrated that DHA can induce ferroptosis in cancer cells by elevating lipid peroxide levels [78,79]. DHA-induced ferroptosis is dependent on the iron-dependent lipoxygenase (LOX) pathway, which is involved in lipid peroxide production. Our study showed a significant increase in lipid peroxidation in MCF-7/Dox cells treated with DHA, suggesting that DHA may activate ferroptosis as a mechanism to sensitize drug-resistant breast cancer cells to the drug.

In the recent past, pyroptosis has emerged as a prospective mechanism by which DHA exerts its anti-cancer properties, particularly in breast cancer. Pyroptosis is a form of programmed cell death that is triggered by inflammatory signals, leading to the release of proinflammatory cytokines and the activation of caspase-1. A recent scoping review by Yurko-Mauro, Van Elswyk et al. (2020), and Newell, Mazurak et al. (2021) examined the influence of the host genetic background on the effectiveness of PUFAs, including DHA, in treating various types of cancer [80,81]. They identified genetic variants involved in inflammation blockade, such as COX-2 activity, PTGS, and CCL2, as well as genes involved in oxidative stress pathways and apoptosis, such as myeloperoxidase genes and NF-kB. A recent study by Pizato, Luzete et al. (2018) demonstrated that DHA can trigger pyroptosis in breast cancer cells [17]. This process is dependent on the NLRP3 inflammasome and the production of reactive oxygen species (ROS). Our research found that DHA treatment led to a significant increase in lipid peroxidation, and pro-apoptotic caspase expression in MCF-7/Dox cells, indicating that DHA may activate pyroptosis to sensitize drug-resistant breast cancer cells to the drug. Additional research is necessary to fully comprehend the activation of ferroptosis and pyroptosis when DHA is used in combination with other treatments for drug-resistant breast cancer cells.

In conclusion, docosahexaenoic acid (DHA) has shown promise as an adjuvant drug to sensitize drug-resistant breast cancer cells to chemotherapy. Based on our findings and those of others, DHA is believed to enhance drug sensitivity through various mechanisms, including modulation of drug efflux pumps; alteration of membrane properties; activation of apoptotic pathways; inhibition of signaling pathways; modulation of epigenetic mechanisms; induction of programmed cell death, including pyroptosis and ferroptosis, in breast cancer cells; and can enhance the effectiveness of chemotherapy drugs such as doxorubicin, docetaxel, and cisplatin. However, further studies are necessary to fully understand the mechanisms of DHA-induced cell death and its potential interactions with other chemotherapy drugs. Clinical trials are also needed to determine the safety and effectiveness of using DHA as a supplement to standard breast cancer treatments. Overall, DHA holds potential as an adjuvant bioactive compound to improve the treatment of drug-resistant breast cancer, but more research is required to establish its efficacy.

## Data Availability

All data generated or analyzed during this study are included in this published article.

## Supplementary Materials

The following supporting information can be downloaded at https://www.mdpi.com/article/10.3390/nu15071658/s1, Figure S1: Anti-proliferative effect of different bioactive fatty acids against parental MCF-7 breast cancer cells. MCF-7 cells were treated with VPA, valproic acid (mM), DHA, docosahexaenoic acid (μM), EPA, eicosapentaenoic acid (μM), 9 HODE (nM), 13 HODE, 13-Hydroxyoctadecadienoic acid (nM), Baicalein (μM), RvD1, Resolvin D1 (nM) and Prot D1, Protectin D1 (nM) for 48 h and percent viability was measured by crystal violet staining. Figure S2: Cytotoxic effect of normal mammary epithelial cell line MCF-10A by MTS assay.

## References

[1] Satsangi A., Roy S.S., Satsangi R.K., Tolcher A.W., Vadlamudi R.K., Goins B., Ong J.L. (2015). Synthesis of a novel, sequentially active-targeted drug delivery nanoplatform for breast cancer therapy. Biomaterials.

[2] Sharmin S., Rahaman M.M., Martorell M., Sastre-Serra J., Sharifi-Rad J., Butnariu M., Bagiu I.C., Bagiu R.V., Islam M.T. (2021). Cytotoxicity of synthetic derivatives against breast cancer and multi-drug resistant breast cancer cell lines: A literature-based perspective study. Cancer Cell Int..

[3] WHO (2021). WHO: Breast Cancer.

[4] Liu H., Liu Y.Z., Zhang F., Wang H.S., Zhang G., Zhou B.H., Zuo Y.L., Cai S.H., Bu X.Z., Du J. (2014). Identification of potential pathways involved in the induction of cell cycle arrest and apoptosis by a new 4-arylidene curcumin analogue T63 in lung cancer cells: A comparative proteomic analysis. Mol. Biosyst..

[5] Favaloro B., Allocati N., Graziano V., Di Ilio C., De Laurenzi V. (2012). Role of apoptosis in disease. Aging.

[6] Glass A.G., Lacey J.V., Jr Carreon J.D., Hoover R.N. (2007). Breast cancer incidence, 1980–2006: Combined roles of menopausal hormone therapy, screening mammography, and estrogen receptor status. J. Natl. Cancer Inst..

[7] Berry D.A., Cronin K.A., Plevritis S.K., Fryback D.G., Clarke L., Zelen M., Mandelblatt J.S., Yakovlev A.Y., Habbema J.D., Feuer E.J. (2005). Effect of screening and adjuvant therapy on mortality from breast cancer. N. Engl. J. Med..

[8] Jemal A., Ward E., Thun M.J. (2007). Recent trends in breast cancer incidence rates by age and tumor characteristics among U.S. women. Breast. Cancer Res..

[9] Liu H., Zang C., Fenner M.H., Possinger K., Elstner E. (2003). PPARgamma ligands and ATRA inhibit the invasion of human breast cancer cells in vitro. Breast. Cancer Res. Treat..

[10] Chavez K.J., Garimella S.V., Lipkowitz S. (2010). Triple negative breast cancer cell lines: One tool in the search for better treatment of triple negative breast cancer. Breast. Dis..

[11] Wyrebska A., Gach K., Lewandowska U., Szewczyk K., Hrabec E., Modranka J., Jakubowski R., Janecki T., Szymanski J., Janecka A. (2013). Anticancer Activity of New Synthetic alpha-Methylene-delta-Lactones on Two Breast Cancer Cell Lines. Basic. Clin. Pharm. Toxicol..

[12] Ali N.M., Yeap S.K., Abu N., Lim K.L., Ky H., Pauzi A.Z.M., Ho W.Y., Tan S.W., Alan-Ong H.K., Zareen S. (2017). Synthetic curcumin derivative DK1 possessed G2/M arrest and induced apoptosis through accumulation of intracellular ROS in MCF-7 breast cancer cells. Cancer Cell Int..

[13] Kheirollahi A., Pordeli M., Safavi M., Mashkouri S., Naimi-Jamal M.R., Ardestani S.K. (2014). Cytotoxic and apoptotic effects of synthetic benzochromene derivatives on human cancer cell lines. Naunyn. Schmiedebergs. Arch Pharm..

[14] Cameron I.L., Munoz J., Barnes C.J., Hardman W.E. (2003). High dietary level of synthetic vitamin E on lipid peroxidation, membrane fatty acid composition and cytotoxicity in breast cancer xenograft and in mouse host tissue. Cancer Cell Int..

[15] Davis D.D., Diaz-Cruz E.S., Landini S., Kim Y.W., Brueggemeier R.W. (2008). Evaluation of synthetic isoflavones on cell proliferation, estrogen receptor binding affinity, and apoptosis in human breast cancer cells. J. Steroid. Biochem. Mol. Biol..

[16] Fabian C.J., Kimler B.F., Hursting S.D. (2015). Omega-3 fatty acids for breast cancer prevention and survivorship. Breast. Cancer Res..

[17] Pizato N., Luzete B.C., Kiffer L., Correa L.H., de Oliveira Santos I., Assumpcao J.A.F., Ito M.K., Magalhaes K.G. (2018). Omega-3 docosahexaenoic acid induces pyroptosis cell death in triple-negative breast cancer cells. Sci. Rep..

[18] Wang T.T., Yang Y., Wang F., Yang W.G., Zhang J.J., Zou Z.Q. (2021). Docosahexaenoic acid monoglyceride induces apoptosis and autophagy in breast cancer cells via lipid peroxidation-mediated endoplasmic reticulum stress. J. Food Sci..

[19] Aslan C., Maralbashi S., Kahroba H., Asadi M., Soltani-Zangbar M.S., Javadian M., Shanehbandi D., Baradaran B., Darabi M., Kazemi T. (2020). Docosahexaenoic acid (DHA) inhibits pro-angiogenic effects of breast cancer cells via down-regulating cellular and exosomal expression of angiogenic genes and microRNAs. Life Sci..

[20] Chen K.M., Thompson H., Vanden-Heuvel J.P., Sun Y.W., Trushin N., Aliaga C., Gowda K., Amin S., Stanley B., Manni A. (2021). Lipoxygenase catalyzed metabolites derived from docosahexaenoic acid are promising antitumor agents against breast cancer. Sci. Rep..

[21] Kuban-Jankowska A., Gorska-Ponikowska M., Sahu K.K., Kostrzewa T., Wozniak M., Tuszynski J. (2019). Docosahexaenoic Acid Inhibits PTP1B Phosphatase and the Viability of MCF-7 Breast Cancer Cells. Nutrients.

[22] Rahman M.M., Veigas J.M., Williams P.J., Fernandes G. (2013). DHA is a more potent inhibitor of breast cancer metastasis to bone and related osteolysis than EPA. Breast. Cancer Res. Treat.

[23] Liscovitch M., Lavie Y. (2002). Cancer multidrug resistance: A review of recent drug discovery research. IDrugs.

[24] Aller S.G., Yu J., Ward A., Weng Y., Chittaboina S., Zhuo R., Harrell P.M., Trinh Y.T., Zhang Q., Urbatsch I.L. (2009). Structure of P-glycoprotein reveals a molecular basis for poly-specific drug binding. Science.

[25] Zahreddine H., Borden K.L. (2013). Mechanisms and insights into drug resistance in cancer. Front. Pharm..

[26] Ling V. (1997). Multidrug resistance: Molecular mechanisms and clinical relevance. Cancer Chemother. Pharm..

[27] Ozben T. (2006). Mechanisms and strategies to overcome multiple drug resistance in cancer. FEBS Lett.

[28] Higgins C.F., Gottesman M.M. (1992). Is the multidrug transporter a flippase?. Trends Biochem. Sci..

[29] Kerbel R.S., Kobayashi H., Graham C.H. (1994). Intrinsic or acquired drug resistance and metastasis: Are they linked phenotypes?. J. Cell Biochem..

[30] Christowitz C., Davis T., Isaacs A., van Niekerk G., Hattingh S., Engelbrecht A.M. (2019). Mechanisms of doxorubicin-induced drug resistance and drug resistant tumour growth in a murine breast tumour model. BMC Cancer.

[31] Conklin K.A. (2004). Chemotherapy-associated oxidative stress: Impact on chemotherapeutic effectiveness. Integr. Cancer Ther..

[32] Sinha B.K. (1989). Free radicals in anticancer drug pharmacology. Chem. Biol. Interact..

[33] Gaba R.C., Emmadi R., Parvinian A., Casadaban L.C. (2016). Correlation of Doxorubicin Delivery and Tumor Necrosis after Drug-eluting Bead Transarterial Chemoembolization of Rabbit VX2 Liver Tumors. Radiology.

[34] Tacar O., Sriamornsak P., Dass C.R. (2013). Doxorubicin: An update on anticancer molecular action, toxicity and novel drug delivery systems. J. Pharm. Pharmcol..

[35] Wei L., Surma M., Gough G., Shi S., Lambert-Cheatham N., Chang J., Shi J. (2015). Dissecting the Mechanisms of Doxorubicin and Oxidative Stress-Induced Cytotoxicity: The Involvement of Actin Cytoskeleton and ROCK1. PLoS ONE.

[36] Chajes V., Sattler W., Stranzl A., Kostner G.M. (1995). Influence of n-3 fatty acids on the growth of human breast cancer cells in vitro: Relationship to peroxides and vitamin-E. Breast Cancer Res. Treat..

[37] Newell M., Patel D., Goruk S., Field C.J. (2020). Docosahexaenoic Acid Incorporation Is Not Affected by Doxorubicin Chemotherapy in either Whole Cell or Lipid Raft Phospholipids of Breast Cancer Cells in vitro and Tumor Phospholipids in vivo. Lipids.

[38] D’Eliseo D., Velotti F. (2016). Omega-3 Fatty Acids and Cancer Cell Cytotoxicity: Implications for Multi-Targeted Cancer Therapy. J. Clin. Med..

[39] Ewaschuk J.B., Newell M., Field C.J. (2012). Docosahexanoic acid improves chemotherapy efficacy by inducing CD95 translocation to lipid rafts in ER(-) breast cancer cells. Lipids.

[40] Newell M., Brun M., Field C.J. (2019). Treatment with DHA Modifies the Response of MDA-MB-231 Breast Cancer Cells and Tumors from nu/nu Mice to Doxorubicin through Apoptosis and Cell Cycle Arrest. J. Nutr..

[41] Newell M., Goruk S., Mazurak V., Postovit L., Field C.J. (2019). Role of docosahexaenoic acid in enhancement of docetaxel action in patient-derived breast cancer xenografts. Breast. Cancer Res. Treat..

[42] Schley P.D., Brindley D.N., Field C.J. (2007). (n-3) PUFA alter raft lipid composition and decrease epidermal growth factor receptor levels in lipid rafts of human breast cancer cells. J. Nutr..

[43] Corsetto P.A., Colombo I., Kopecka J., Rizzo A.M., Riganti C. (2017). Omega-3 Long Chain Polyunsaturated Fatty Acids as Sensitizing Agents and Multidrug Resistance Revertants in Cancer Therapy. Int. J. Mol. Sci..

[44] Germain E., Chajes V., Cognault S., Lhuillery C., Bougnoux P. (1998). Enhancement of doxorubicin cytotoxicity by polyunsaturated fatty acids in the human breast tumor cell line MDA-MB-231: Relationship to lipid peroxidation. Int. J. Cancer.

[45] Menendez J.A., Lupu R., Colomer R. (2005). Exogenous supplementation with omega-3 polyunsaturated fatty acid docosahexaenoic acid (DHA; 22:6n-3) synergistically enhances taxane cytotoxicity and downregulates Her-2/neu (c-erbB-2) oncogene expression in human breast cancer cells. Eur. J. Cancer Prev..

[46] Kang K.S., Wang P., Yamabe N., Fukui M., Jay T., Zhu B.T. (2010). Docosahexaenoic acid induces apoptosis in MCF-7 cells in vitro and in vivo via reactive oxygen species formation and caspase 8 activation. PLoS ONE.

[47] Bougnoux P., Hajjaji N., Ferrasson M.N., Giraudeau B., Couet C., Le Floch O. (2009). Improving outcome of chemotherapy of metastatic breast cancer by docosahexaenoic acid: A phase II trial. Br. J. Cancer.

[48] He M., Guo S., Li Z. (2015). In situ characterizing membrane lipid phenotype of breast cancer cells using mass spectrometry profiling. Sci. Rep..

[49] Stillwell W., Shaikh S.R., Zerouga M., Siddiqui R., Wassall S.R. (2005). Docosahexaenoic acid affects cell signaling by altering lipid rafts. Reprod. Nutr. Dev..

[50] Fodil M., Blanckaert V., Ulmann L., Mimouni V., Chenais B. (2022). Contribution of n-3 Long-Chain Polyunsaturated Fatty Acids to the Prevention of Breast Cancer Risk Factors. Int. J. Environ. Res. Public Health.

[51] Chen X., Li W., Ren J., Huang D., He W.T., Song Y., Yang C., Li W., Zheng X., Chen P. (2014). Translocation of mixed lineage kinase domain-like protein to plasma membrane leads to necrotic cell death. Cell Res..

[52] Turk H.F., Chapkin R.S. (2013). Membrane lipid raft organization is uniquely modified by n-3 polyunsaturated fatty acids. Prostaglandins. Leukot. Essent. Fat. Acids..

[53] Biondo P.D., Brindley D.N., Sawyer M.B., Field C.J. (2008). The potential for treatment with dietary long-chain polyunsaturated n-3 fatty acids during chemotherapy. J. Nutr. Biochem..

[54] Rogers K.R., Kikawa K.D., Mouradian M., Hernandez K., McKinnon K.M., Ahwah S.M., Pardini R.S. (2010). Docosahexaenoic acid alters epidermal growth factor receptor-related signaling by disrupting its lipid raft association. Carcinogenesis.

[55] Chapkin R.S., Wang N., Fan Y.Y., Lupton J.R., Prior I.A. (2008). Docosahexaenoic acid alters the size and distribution of cell surface microdomains. Biochim. Biophys. Acta.

[56] Kim W., Fan Y.Y., Barhoumi R., Smith R., McMurray D.N., Chapkin R.S. (2008). n-3 polyunsaturated fatty acids suppress the localization and activation of signaling proteins at the immunological synapse in murine CD4+ T cells by affecting lipid raft formation. J. Immunol..

[57] Kauffman M.K., Kauffman M.E., Zhu H., Jia Z., Li Y.R. (2016). Fluorescence-Based Assays for Measuring Doxorubicin in Biological Systems. React. Oxyg. Species.

[58] Brown I., Lee J., Sneddon A.A., Cascio M.G., Pertwee R.G., Wahle K.W.J., Rotondo D., Heys S.D. (2020). Anticancer effects of n-3 EPA and DHA and their endocannabinoid derivatives on breast cancer cell growth and invasion. Prostaglandins. Leukot. Essent. Fat. Acids.

[59] Baumgartner M., Sturlan S., Roth E., Wessner B., Bachleitner-Hofmann T. (2004). Enhancement of arsenic trioxide-mediated apoptosis using docosahexaenoic acid in arsenic trioxide-resistant solid tumor cells. Int. J. Cancer.

[60] Chauvin L., Goupille C., Blanc C., Pinault M., Domingo I., Guimaraes C., Bougnoux P., Chevalier S., Maheo K. (2016). Long chain n-3 polyunsaturated fatty acids increase the efficacy of docetaxel in mammary cancer cells by downregulating Akt and PKCepsilon/delta-induced ERK pathways. Biochim. Biophys. Acta.

[61] Vibet S., Goupille C., Bougnoux P., Steghens J.P., Gore J., Maheo K. (2008). Sensitization by docosahexaenoic acid (DHA) of breast cancer cells to anthracyclines through loss of glutathione peroxidase (GPx1) response. Free Radic. Biol. Med..

[62] Lindskog M., Gleissman H., Ponthan F., Castro J., Kogner P., Johnsen J.I. (2006). Neuroblastoma cell death in response to docosahexaenoic acid: Sensitization to chemotherapy and arsenic-induced oxidative stress. Int. J. Cancer.

[63] Liu X., Sun C., Jin X., Li P., Ye F., Zhao T., Gong L., Li Q. (2013). Genistein enhances the radiosensitivity of breast cancer cells via G(2)/M cell cycle arrest and apoptosis. Molecules.

[64] Kimani S., Chakraborty S., Irene I., de la Mare J., Edkins A., du Toit A., Loos B., Blanckenberg A., Van Niekerk A., Costa-Lotufo L.V. (2021). The palladacycle, BTC2, exhibits anti-breast cancer and breast cancer stem cell activity. Biochem. Pharm..

[65] Gopisetty M.K., Adamecz D.I., Nagy F.I., Baji A., Lathira V., Szabo M.R., Gaspar R., Csont T., Frank E., Kiricsi M. (2021). Androstano-arylpyrimidines: Novel small molecule inhibitors of MDR1 for sensitizing multidrug-resistant breast cancer cells. Eur. J. Pharm. Sci..

[66] Shinde A., Kulkoyluoglu Cotul E., Chen H., Smith A., Libring S., Solorio L., Wendt M.K. (2022). Transglutaminase-2 mediates acquisition of neratinib resistance in metastatic breast cancer. Mol. Biomed..

[67] Wu C.P., Hung C.Y., Murakami M., Wu Y.S., Lin C.L., Huang Y.H., Hung T.H., Yu J.S., Ambudkar S.V. (2022). P-glycoprotein Mediates Resistance to the Anaplastic Lymphoma Kinase Inhiitor Ensartinib in Cancer Cells. Cancers.

[68] Herman J.F., Mangala L.S., Mehta K. (2006). Implications of increased tissue transglutaminase (TG2) expression in drug-resistant breast cancer (MCF-7) cells. Oncogene.

[69] Cheng K., Wang X.H., Hua Y.T., Zhang Y.Z., Han Y., Yang Z.L. (2020). The tissue transglutaminase: A potential target regulating MDR in breast cancer. Eur. Rev. Med. Pharm. Sci..

[70] Cabaud O., Berger L., Crompot E., Adelaide J., Finetti P., Garnier S., Guille A., Carbuccia N., Farina A., Agavnian E. (2022). Overcoming Resistance to Anti-Nectin-4 Antibody-Drug Conjugate. Mol. Cancer Ther..

[71] Robinson K., Tiriveedhi V. (2020). Perplexing Role of P-Glycoprotein in Tumor Microenvironment. Front Oncol..

[72] Kumar A., Jaitak V. (2019). Natural products as multidrug resistance modulators in cancer. Eur. J. Med. Chem..

[73] Wang F., Lv P., Gu Y., Li L., Ge X., Guo G. (2017). Galectin-1 knockdown improves drug sensitivity of breast cancer by reducing P-glycoprotein expression through inhibiting the Raf-1/AP-1 signaling pathway. Oncotarget.

[74] Kuan C.Y., Walker T.H., Luo P.G., Chen C.F. (2011). Long-chain polyunsaturated fatty acids promote paclitaxel cytotoxicity via inhibition of the MDR1 gene in the human colon cancer Caco-2 cell line. J. Am. Coll. Nutr..

[75] Maheo K., Vibet S., Steghens J.P., Dartigeas C., Lehman M., Bougnoux P., Gore J. (2005). Differential sensitization of cancer cells to doxorubicin by DHA: A role for lipoperoxidation. Free Radic. Biol. Med..

[76] Vermonden P., Vancoppenolle M., Dierge E., Mignolet E., Cuvelier G., Knoops B., Page M., Debier C., Feron O., Larondelle Y. (2021). Punicic Acid Triggers Ferroptotic Cell Death in Carcinoma Cells. Nutrients.

[77] Dierge E., Debock E., Guilbaud C., Corbet C., Mignolet E., Mignard L., Bastien E., Dessy C., Larondelle Y., Feron O. (2021). Peroxidation of n-3 and n-6 polyunsaturated fatty acids in the acidic tumor environment leads to ferroptosis-mediated anticancer effects. Cell Metab..

[78] Zhang Z., Wang X., Wang Z., Zhang Z., Cao Y., Wei Z., Shao J., Chen A., Zhang F., Zheng S. (2021). Dihydroartemisinin alleviates hepatic fibrosis through inducing ferroptosis in hepatic stellate cells. Biofactors.

[79] Shan K., Feng N., Zhu D., Qu H., Fu G., Li J., Cui J., Chen H., Wang R., Qi Y. (2022). Free docosahexaenoic acid promotes ferroptotic cell death via lipoxygenase dependent and independent pathways in cancer cells. Eur. J. Nutr..

[80] Yurko-Mauro K., Van Elswyk M., Teo L. (2020). A Scoping Review of Interactions between Omega-3 Long-Chain Polyunsaturated Fatty Acids and Genetic Variation in Relation to Cancer Risk. Nutrients.

[81] Newell M., Mazurak V., Postovit L.M., Field C.J. (2021). N-3 Long-Chain Polyunsaturated Fatty Acids, Eicosapentaenoic and Docosahexaenoic Acid, and the Role of Supplementation during Cancer Treatment: A Scoping Review of Current Clinical Evidence. Cancers.

