# Supplementary material for: Docosahexaenoic Acid, a Key Compound for Enhancing Sensitization to Drug in Doxorubicin-Resistant MCF-7 Cell Line
Source: Nutrients. 2023 Mar 29;15(7):1658. doi: 10.3390/nu15071658 (PMC10097357; doi:10.3390/nu15071658)
Supplement: Supplementary file 1 [file nutrients-15-01658-s001.zip › nutrients-2246082-supplementary.pdf]

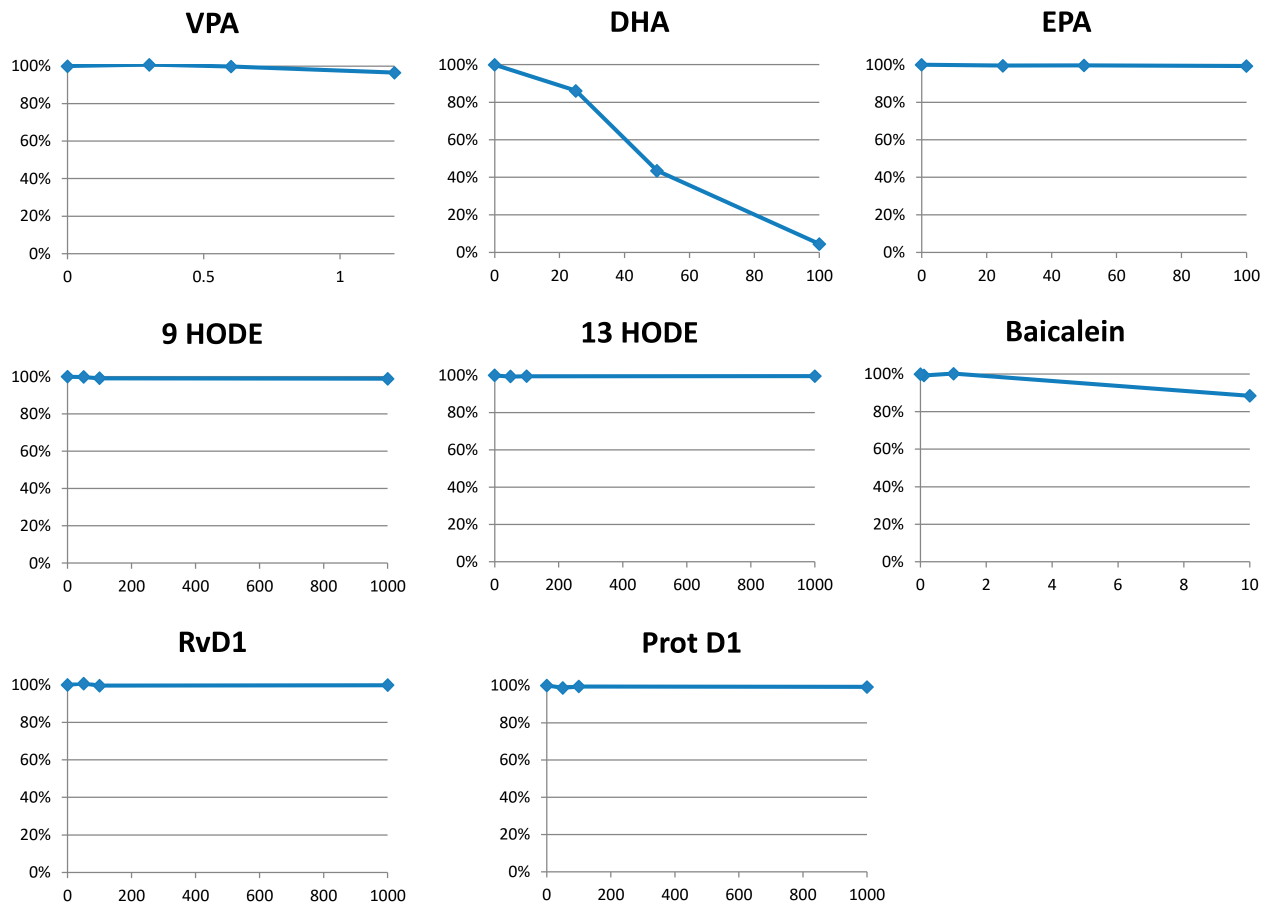

Figure S1. Anti-proliferative effect of different bioactive fatty acids against parental MCF-7 breast cancer cells. MCF-7 cells were treated with VPA, valproic acid (mM), DHA, docosahexaenoic acid (uM), EPA, eicosapentaenoic acid (uM), 9 HODE (nM), 13 HODE, 13-Hydroxyoctadecadienoic acid (nM), Baicalein (uM), RvD1, Resolvin D1 (nM) and Prot D1, Protectin D1 (nM) for 48h and percent viability was measured by crystal violet staining.

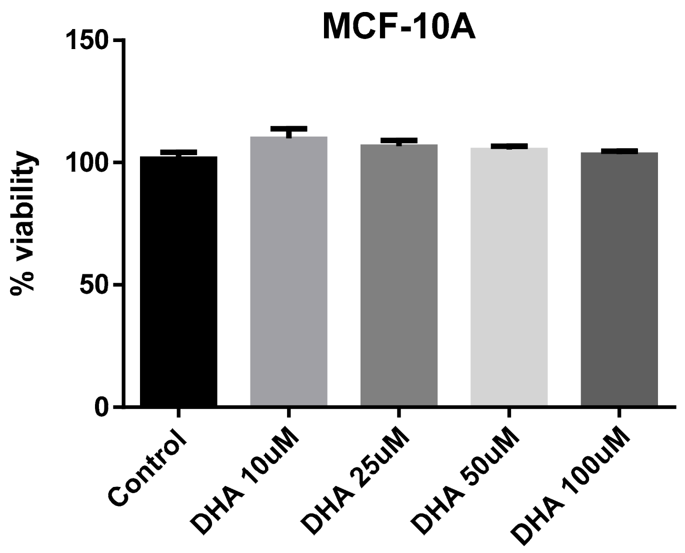

Figure S2: Cytotoxic effect of normal mammary epithelial cell line MCF-10A by MTS assay.
